# A Review of Machine Learning Algorithms for Retinal Cyst Segmentation on Optical Coherence Tomography

**DOI:** 10.3390/s23063144

**Published:** 2023-03-15

**Authors:** Xing Wei, Ruifang Sui

**Affiliations:** Department of Ophthalmology, State Key Laboratory of Complex Severe and Rare Diseases, Peking Union Medical College Hospital, Chinese Academy of Medical Sciences, Peking Union Medical College, Beijing 100730, China; weixingcocoa@163.com

**Keywords:** cyst segmentation, optical coherence tomography, machine learning, deep learning

## Abstract

Optical coherence tomography (OCT) is an emerging imaging technique for diagnosing ophthalmic diseases and the visual analysis of retinal structure changes, such as exudates, cysts, and fluid. In recent years, researchers have increasingly focused on applying machine learning algorithms, including classical machine learning and deep learning methods, to automate retinal cysts/fluid segmentation. These automated techniques can provide ophthalmologists with valuable tools for improved interpretation and quantification of retinal features, leading to more accurate diagnosis and informed treatment decisions for retinal diseases. This review summarized the state-of-the-art algorithms for the three essential steps of cyst/fluid segmentation: image denoising, layer segmentation, and cyst/fluid segmentation, while emphasizing the significance of machine learning techniques. Additionally, we provided a summary of the publicly available OCT datasets for cyst/fluid segmentation. Furthermore, the challenges, opportunities, and future directions of artificial intelligence (AI) in OCT cyst segmentation are discussed. This review is intended to summarize the key parameters for the development of a cyst/fluid segmentation system and the design of novel segmentation algorithms and has the potential to serve as a valuable resource for imaging researchers in the development of assessment systems related to ocular diseases exhibiting cyst/fluid in OCT imaging.

## 1. Introduction

Optical coherence tomography (OCT) is a non-invasive high-resolution imaging technique that enables the visualizing of the characteristics of biological samples [1]. It was widely applied for diagnostic and prognostic of retinal disease, particularly in the analysis of conditions that have an impact on the microstructure of the retina, including retinal layers, exudates, cysts, and fluid [2]. Retinal cyst/fluid is a fluid-filled space in the retina and is a pathological consequence of several common ocular diseases, including age-related macular degeneration (AMD), retinal vein occlusion (RVO), diabetic macular edema (DME), ocular inflammation, and diabetic retinopathy (DR) [3,4,5,6]. The detection, location, and extent of cystoid macular edema (CME) served as indicators of disease progression and aided in patient-specific treatment planning and prognosis evaluation. For instance, several medical studies have established that visual acuity can be estimated from the volume of retinal fluid in the OCT images of diabetes and uveitis patients with CME [7,8].

**Figure 1:**
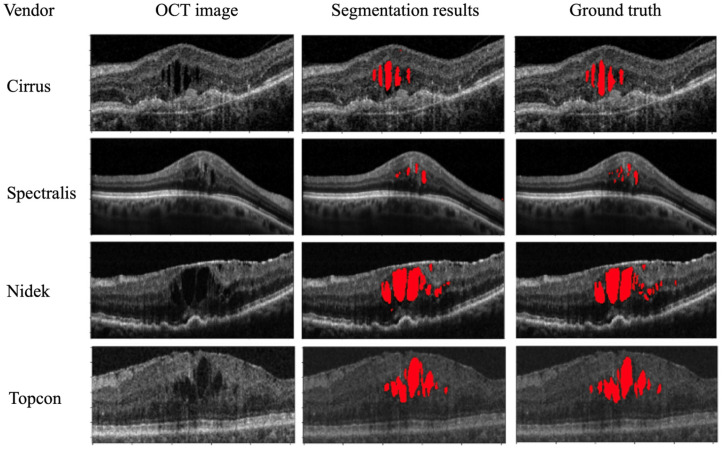
Illustration of cyst/fluid regions and segmentation results in OCT images [9]. The OCT images were acquired with scanners from various vendors, including Topocon, Cirrus, Spectralis, and Nidek. The first column of subfigures shows the OCT images, the red region in the second column shows the algorithm’s automated segmentation results, and red region in the third column shows the expert-annotated ground truth of the cyst/fluid regions.

However, manual segmentation of cyst/fluid in OCT images, which requires retinal expert identification, is prone to deviations in subjective interpretation and poor repeatability. Additionally, the large amount of imaging data per patient makes manual assessment extremely time-consuming. These challenges have motivated lots of researchers to propose automated algorithms for cyst segmentation/measurement to speed up the process and reduce human labor. Automated segmentation techniques to the cysts/fluid can provide ophthalmologists with valuable tools for improved interpretation and evaluation of retinal features, which is helpful for more accurate diagnoses and guided intervention. An illustration of cyst/fluid segmentation is shown in Figure 1. The cysts were detected by the segmentation algorithm [9] and annotated as red.

Recently, artificial intelligence (AI) has experienced significant growth with the advent of deep learning, offering a better representation of data [10]. These advances have resulted in major breakthroughs in the application of AI to natural language processing and computer vision areas that previously could be conducted only by humans [11,12,13]. Machine learning can generally be categorized into two categories: classical machine learning and deep learning. Classical machine learning algorithms offer flexibility and interpretability, while deep learning algorithms provide powerful scalability and hierarchical feature learning capabilities that can automatically extract morphological features from raw image data. For image processing of OCT data, deep learning algorithms have made significant strides in the segmentation of retinal cyst/ fluids, with popular frameworks, including convolutional neural network (CNN), fully convolutional network (FCN), and U-shape network (U-Net). Meanwhile, with the increasing volume of data available in clinical research, there are huge opportunities to apply AI techniques to improve disease diagnosis and patient care [14]. Furthermore, because of the image-centric nature of ophthalmology, where diagnosis often hinges on image analysis through techniques, such as fundus and OCT imaging, ophthalmology has become one of the frontiers of AI research.

The process of cyst segmentation can be broken down into three distinct steps, namely image denoising, retinal layer segmentation, and cyst/fluid segmentation. The initial image denoising step serves to eliminate speckle noise and enhance edge information in OCT images. Retinal layer segmentation identifies potential regions where cysts or fluids may exist, reducing the possibility of false positive errors in subsequent steps. Finally, the cyst/fluid segmentation step identifies the precise location of each cyst or fluid region within the OCT images. In a cyst segmentation system, all three of these steps are used to-gather to accurately and efficiently identify and segment cystic structures in OCT images.

**Figure 2:**
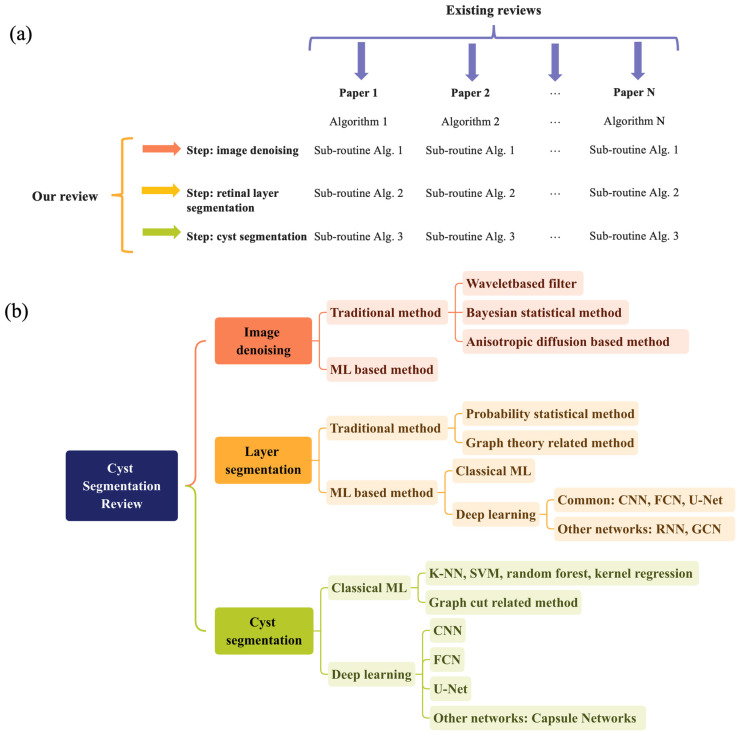
The perceptive and the structure of our review: (**a**) the perspective of our review on the cyst segmentation problem compared with the existing reviews; (**b**) the structure of our review on the cyst segmentation problem.

In this paper, we review existing works for retinal cyst/fluid segmentation in OCT images, with an emphasis on the importance of machine learning techniques. Given the critical role of image denoising and retinal layer segmentation in many cyst/fluid segmentation algorithms, we also review the state-of-the-art methods in these two areas and discuss their advantages and disadvantages. This review is distinct from other related works in the field, which focus only on the core cyst/fluid segmentation algorithm. We provide a holistic overview of each component of the cyst segmentation system, including image denoising, retinal layer segmentation, and cyst/fluid segmentation, as depicted in Figure 2.

The main contributions of our study can be summarized as follows: Firstly, we present a comprehensive overview of the current state of retinal cyst segmentation in optical OCT and highlight the key findings of machine learning techniques. Secondly, we provide a summary of the publicly available OCT datasets for cyst/fluid segmentation. Thirdly, we discuss the challenges, opportunities, and future directions for the application of machine learning in retinal cyst segmentation. Overall, this review provides valuable insight into the development of new segmentation algorithms and the improvement of existing ones.

## 2. Cyst Segmentation System

The process of the cyst segmentation system can be divided into three steps, which includes image denoising, retinal layer segmentation, and cyst/fluid segmentation. The image denoising step removes speckle noise and enhances the edge information in the OCT images. The retinal layer segmentation step identifies the potential regions where fluid or cysts may exist and reduces the likelihood of false positive errors in the subsequent step. The cyst/fluid segmentation step finally locates the position of each cyst or fluid region in the OCT images.

### 2.1. Denoising Technique of Image

The limited spatial bandwidth of the interference signals in OCT imaging lead to the occurrence of speckle noise, which significantly hinders subsequent analysis and segmentation in OCT. The dual role of speckle noise as both a source of noise and a carrier of information regarding tissue structure presents challenges in accurately identifying the boundaries of retinal layers and other retinal features in OCT, such as cysts/fluids. To address these difficulties, a number of techniques for speckle denoising have been proposed in the literature, including spatially adaptive wavelet filter [15], Bayesian least-squares estimation [16], anisotropic diffusion filters [17,18,19,20,21], a combination of bilateral and median filtering [19,22], and a machine learning based method [23]. In this section, we evaluate the most common speckle denoising methods in OCT images. These methods have been extensively studied and have demonstrated their effectiveness in reducing speckle noise while preserving important structure information.

The spatially adaptive wavelet filter (SAWF) [15] is a wavelet-based algorithm designed to alleviate speckle noise in both time-domain and Fourier-domain OCT images. The SAWF separates the edges in OCT and suppresses the noise power in the wavelet domain while preserving the sharpness of the tissue. The algorithm has been tested in OCT and shown to improve the signal-to-noise ratio more than 7 dB with a minimal reduction in sharpness of 3% or less. Despite the advantages of wavelet-based methods, such as reduced speckle noise and preserved detail in comparison to conventional techniques, these methods can also generate image artefacts related to the wavelet used. These image artefacts negatively impact the visibility of delicate structural details and consequently compromise the overall quality of OCT images. Alternative works according to spatial compounding [15] or frequency compounding [24] have also been proposed, which leverage the uncorrelated nature of speckle patterns at different positions, angles, and wavelengths to enhance image contrast. These methods involve obtaining several scans of the same sample at varying angles and then combining the data to reduce speckle noise. Studies have indicated that this technique effectively reduces speckle noise and improves the quality of OCT images. However, in cases where strong noise reduction is required, these techniques may result in substantial blurring of edges [25].

Wong et al. [16] presented an algorithm to reduce speckle noise in OCT according to Bayesian statistical techniques. The approach modelled the speckle noise and estimated the true underlying signal, resulting in improved speckle reduction and preservation of image details according to the experiments. Another study by Fang et al. [26] proposed a sparsity-based denoising method for OCT images named multiscale sparsity-based tomographic denoising (MSBTD). This method involves creating a sparse representation dictionary of OCT images with high signal-to-noise ratios (SNRs), which can then be used to denoise OCT images with low SNRs. MSBTD has been implemented on the Duke database and shown to effectively reduce noise in OCT images while maintaining the overall image quality.

The use of anisotropic diffusion-based methods [19,20,21] has increased in popularity in recent years for the reduction of speckle noise. As compared to wavelet-based methods, they improve noise suppression, reduce detail loss, and do not introduce artifacts into the processed images. However, under high speckle noise contamination, their performance of speckle noise suppression is limited.

Taking advantage of recent developments in deep learning, various learning-based approaches have been put forth to tackle the problem of speckle noise in OCT images. Saba et al. [23] introduced the learnable de-speckling framework (LDF) which employed a figure of merit (FOM) as a quantitative metric for identifying the most effective method for reducing speckle noise in OCT images. The LDF comprised two components: an autoencoder neural network and a filter classifier. The former was responsible for learning the FOM by evaluating multiple quality assessment metrics derived from the OCT image, while the latter selected the most efficient filter from a variety of options, including sliding window filters, adaptive statistical-based filters, and edge-preserving filters based on patch or pixel correlation.

### 2.2. Retinal Layer Segmentation

**Figure 3:**
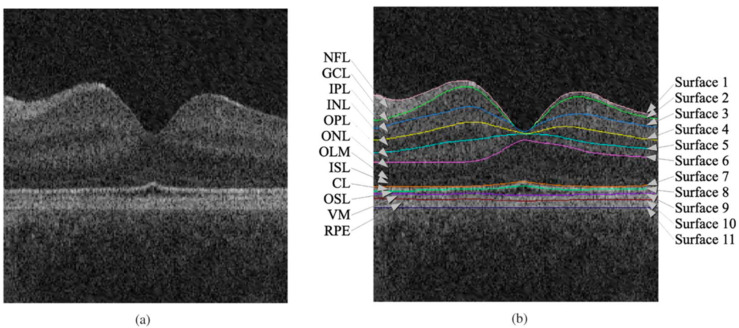
Segmentation result of 11 retinal surfaces (10 layers) of an OCT image: (**a**) OCT image. (**b**) layer segmentation results, nerve fiber layer (NFL), ganglion cell layer (GCL), inner plexiform layer (IPL), inner nuclear layer (INL), outer plexiform layer (OPL), outer nuclear layer (ONL), outer limiting membrane (OLM), inner segment layer (ISL), connecting cilia (CL), outer segment layer (OSL), Verhoeff’s membrane (VM), and retinal pigment epithelium (RPE). The figure was reprinted with permission from Ref. [27]. 2010, Queelec.

Retinal layer segmentation in OCT is a critical pre-processing step in retinal cyst/fluid segmentation, as illustrated in Figure 3. The location of different types of cysts/fluids in the OCT images varies depending on the underlying disease. Layer segmentation provides a constraint for the region of the cyst segmentation search, thus improving detection accuracy. This step is crucial as the cyst/fluid in OCT images often appear dark and indistinguishable from the “background”. It is essential to identify and eliminate locations that are not the cyst/fluid by leveraging prior knowledge of the doctor, which decreases the false positive error in the cyst segmentation. Researchers have proposed two main categories of methods for retinal layer segmentation, traditional methods and machine learning methods.

Based on statistical models, traditional methods for segmenting retinal layers in OCT images involve partitioning the image into homogeneous segments according to characteristics, such as intensity or texture. For instance, Hee et al. segmented the entire retinal layer and the nerve fiber layer by the variations of pixel gray value of pixels [28]. Traditional thresholding techniques are prone to errors because of the sensitivity to noise and intensity inhomogeneities and overlooking the significance of the spatial information in OCT images. To address this limitation, Herzog et al. introduced a method that incorporates edge maximization and smoothness constraints to determine an optimal threshold for automatically extracting the optic nerve and retinal boundaries [29]. The algorithm considered the maximization of edges located on the boundary and the minimization of the boundary’s average rate of change, thereby ensuring accurate identification of the vitreal–retinal boundary in OCT images.

Retinal layer segmentation in OCT images has also been investigated in deformable-model-based methods. Lu et al. formulated the OCT datasets as probability density fields and applied a level set model to outline the retinal nerve fiber layer [30]. More specifically, the approach defined region boundaries by utilizing the symmetrized Kullback–Leibler distance. The algorithm can be seen as a curve evolution model, as it outlined object boundaries in images through iterative relaxation of a closed curve or surface. Likewise, the level set method was also applied by Wang et al. [31] for the segmentation of the retinal nerve fiber layer. In detail, their approach began by eliminating OCT imaging artefacts, including speckle noise, using 3D nonlinear anisotropic filtering to enhance the contrast between layers. Subsequently, the level set method, the k-means method, and the Markov random field methods were employed for the purpose of segmenting the three inner retinal layers around the optic nerve head, thereby effectively overcoming the presence of artefacts, such as blood vessel shadows and variations in image intensity.

Graph-cut-based algorithms have been explored for layer segmentation in OCT. Garvin et al. introduced a graph-theoretic segmentation approach that optimized the simultaneous segmentation of multiple 3D surfaces through the application of the minimum cut algorithm in a graph [32]. This method enabled the consideration of different feasibility constraints and the integration of accurate regional information. Their method had the ability to incorporate varying feasibility constraints and the ability to incorporate true regional information. Similarly, Chiu et al. also applied graph theory and dynamic programming techniques to segment eight boundaries of retinal layers, leading to a substantial reduction in the processing time of the algorithm [33]. Gao et al. presented a novel approach to OCT layer segmentation by formulating the images as weighted graphs, placing emphasis on super-pixels and connected component-based image cues. This method effectively addressed the challenges posed by speckle noise, organic textures, and blood vessel artefacts. The proposed algorithm is accurate and efficient, providing reliable quantification of layer boundaries [34].

In the realm of classical machine learning, Karri et al. presented a structured random forest to detect eight retinal layer boundaries employing traditional graph theory for AMD [35]. The method resolved the issue of the missing edge by correcting the prediction using neighboring predictions. Recently, multiple deep-learning-based image segmentation methods have been designed for segmenting OCT layers. Fang et al. developed a hybrid approach (named CNN-GS) for retinal layer segmentation, combining a modified convolutional neural network (CNN) and a graph search method. The effectiveness of this approach was evaluated using a dataset of AMD patients [32]. The CNN was utilized to extract features of the retinal layer boundaries and produce a preliminary estimate of the eight layers, while the graph search method utilized the resulting probability maps to identify the final layer boundaries [36]. Alternatively, Kugelman employed a recurrent neural network (RNN) combined with a graph search method (RNN-GS) for the automatic segmentation of OCT layers. The proposed approach was capable of accurately and consistently segmenting seven retinal layer boundaries in OCT images from healthy individuals and three retinal layer boundaries in OCT images from patients with AMD. Their study indicated that the RNN-GS performed competitively against a previously CNN-based method (CNN-GS) [37]. The layer segmentation using FCN was also exploited. He et al. employed FCN to directly formulate the distribution of surface positions in order to obtain smooth, continuous surfaces in a single feed-forward operation. During the training and testing stages of the deep network, a special topology module was applied to ensure that the surface topology was preserved. An evaluation of the method was conducted on a dataset of healthy individuals, individuals with multiple sclerosis (MS), and individuals with AMD [38]. Furthermore, to address the difficulties posed by the multi-scale features of the optic disc and retinal layers with diverse thicknesses in OCT layer segmentation, Li developed a two-stage framework utilizing a graph convolutional network (GCN), allowing the optic disc and retinal layers to be detected sequentially [39].

### 2.3. Cyst/Fluid Segmentation

The segmentation of cysts/fluid in OCT images is still challenging, even after the application of denoising and layer segmentation techniques. This was due to several factors that complicate the efficacy of conventional segmentation algorithms. The intensity patterns observed in these images are a result of light absorption and scattering in the retinal tissue, leading to a decrease in the intensity of homogeneous areas with increasing depth. This made it difficult for segmentation algorithms that rely solely on intensity variations to accurately segment the cysts/fluid. The presence of low optical contrast in certain regions of the OCT images caused by optical shadowing of retinal blood vessels further exacerbates the problem. Additionally, motion artefacts and sub-optimal imaging conditions negatively impacted the accuracy of segmentation techniques.

To address these challenges, various algorithms for cyst/fluid segmentation in OCT images have been proposed and can be grouped into two main categories: classical machine learning methods and deep-neural-network-based learning methods.

#### 2.3.1. Classical Machine Learning Methods

Classical machine learning is a branch of machine learning that encompasses traditional algorithms that were developed prior to the advent of deep learning. These algorithms are based on mathematical and statistical models and are widely used for various tasks, including regression and classification. The most common classical machine learning algorithms include K-nearest neighbor (k-NN), support vector machine (SVM), random forest, kernel-based regression, graph search-graph cut method, and AdaBoost. K-NN is a simple and intuitive algorithm that classifies a new data point based on the classes of its k nearest neighbors [40]. SVM is a supervised algorithm that seeks to find the best boundary between different classes of data by maximizing the margin between them [41]. Random forest is an ensemble method that combines multiple decision trees to produce a more accurate prediction [42]. Kernel-based regression uses a kernel function to map the input data into a high-dimensional feature space where linear regression can be applied, allowing for the modeling of complex non-linear relationships between the features and the target variable [43]. The graph-theory-based search and cut method uses graph theory to detect and segment objects in images [44]. AdaBoost is an approach for binary classification problems that combines weak classifiers into a strong classifier by adjusting the sample weights based on the accuracy of the previous classifier [45].

**Figure 4:**
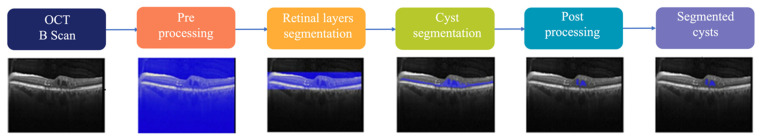
Workflow of classical machine learning in OCT cyst segmentation problem. The OCT image was reprinted with permission from Ref. [46]. 2020, Ganjee.

The majority of classical machine-learning-based works in the field of cyst segmentation adopt a simple classification approach that leverages hand-crafted or texture-based features. The typical framework of these methods consists of four stages, including pre-processing, retinal layer segmentation, cyst segmentation, and post-processing [46] (as illustrated in Figure 4). The objective of the pre-processing stage is to improve and normalize the OCT images, while the retinal layer segmentation aims to locate the relevant regions of interest. The post-processing stage is implemented to reduce the number of false positive regions, that is, regions that are mistakenly segmented as cysts. The typical classical machine learning methods are summarized in Table 1.

Initially, due to the limitations of data and computational power, simple methods were employed by researchers in the field of classical machine learning for cyst segmentation. In 2010, Quellec et al. [27] proposed an automated approach for the three-dimensional analysis of retina texture and quantification of fluid-filled regions associated with AMD. The core segmentation algorithm is derived from the K-nearest neighbor classifier. In pre-processing, the wavelet transform has been widely used in OCT images for denoising and de-speckling [15,57]. A multiscale 3-D graph search technique [47] was used for layer segmentation. Cyst candidates were initially detected using center-surround difference in a multiscale setting and then classified based on statistical texture features, including intensity-level distribution measures, run length measures, co-occurrence matrix measures, and wavelet analysis measures. In 2013, González et al. [48] applied naïve Bayes, SVM, and random forest to 30 high-definition OCT gray-scale images from DR patients. They adapted Haeker’s method [58] to detect the internal limiting membrane and retinal pigment epithelium and a watershed algorithm [59] to find dark areas in the image as cyst candidates. These candidate regions were then filtered by eliminating regions that were too small, elongated, not dense enough, or close to the image sides or boundary top layer. The remaining regions were classified according to three classic machine learning methods using co-occurrence and Gabor filter features. The SVM achieved an accuracy of 80%.

Recently, the increased involvement of researchers in the field of OCT segmentation has led to the publication of several annotated OCT datasets. In 2015, the MICCAI conference hosted the OPTIMA Cyst Segmentation Challenge, releasing an OPTIMA dataset comprising 30 volumes from four different OCT devices used in ophthalmology (Spectralis, Topcon, Nidek, and Cirrus). On the basis of this dataset, Gopinath et al. [49] proposed random forests for classifying detected regions into cystic regions. In their study, the OCT image was first standardized for size and degraded by speckle noise through total variational denoising [50]. The retinal layers were segmented using a graph-theory-based algorithm [33]. The candidate cystic regions were located by the center-surround difference method [51]. Finally, random forest was applied to segment the cyst using the texture of the pattern [60]. In the same year, an annotated segmentation dataset of images of eyes with DME was published by Duke University. Chiu et al. kernel regression for the segmentation problem [52]. The Spectralis registration boundaries were removed, and the images were standardized by the DME algorithm [53,54]. The ILM and BM were detected through GTDP. Before conducting kernel regression, KR-based denoising was employed.

The application of graph theory in image segmentation has gained significant attention in the field due to its ability to effectively represent the relationships between pixels or voxels in an image. Chen et al. [55] investigated segmentation problems for patients with exudative AMD, utilizing a graph search-graph cut (GS-GC) classifier. Specifically, their approach involved Gavin’s algorithm in detecting retinal layer boundaries [32], followed by using probability normalization to refine the initial results. For intra-retinal CME segmentation, a SNR balancing [56] was performed on each OCT scan to obtain a uniform intensity profile. The method isolated macular hole (MH) and vessel silhouettes before extracting volumetric features of the CME. An improved region-growing method was applied to delineate the MH using its detected footprint, while vessel silhouettes were effectively detected using a vessel detector [61]. Finally, AdaBoost was combined with the GS-GC method to segment the CME regions.

#### 2.3.2. Deep-Learning-Based Segmentation Methods

**Figure 5:**
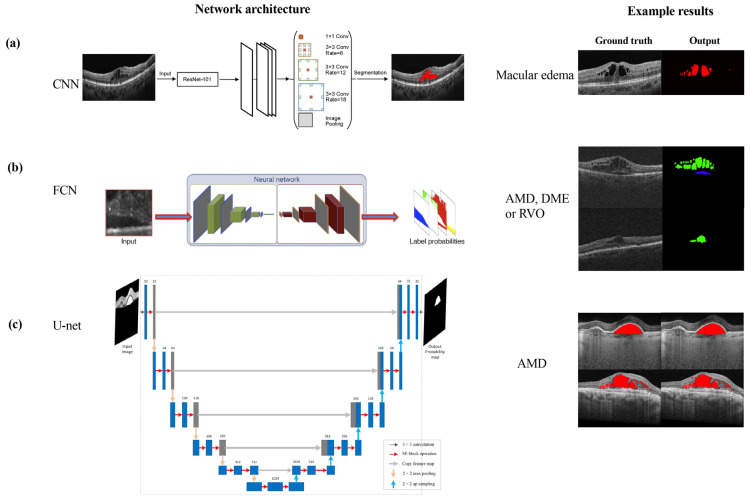
Comparison of three different neural network structures and their respective outcomes regarding segmentation results. The first structure, represented in (**a**), is a CNN model specifically designed for OCT analysis in patients with macular edema [63]. The second structure, depicted in (**b**), is another FCN-based model, which is utilized for OCT analysis in patients suffering from AMD, DME, or RVO. Reprinted with permission from Ref. [64]. 2018, Schlegl.; (**c**) presents a U-net based model, which is specifically designed for OCT analysis of patients with AMD. Reprinted with permission from Ref. [72]. 2016, Wu. The colored regions in the right side images are the segmentation results.

The utilization of deep learning for automated segmentation in the field of medical imaging has garnered substantial interest. Compared with classical methods, these methods need a more extensive dataset, do not require human-engineered features, and simplify the system design by training an end-to-end classifier. Deep neural networks simplify the entire system design, as a single neural network is capable of detecting retinal-layer-bound cysts and segmenting cysts. Table 2 provides a concise overview of deep-neural-network-based learning methods, while Table 3 presents a summary of the performance metrics of these methods. The majority of current works are based on convolutional neural networks (CNNs) or their modifications, such as fully convolutional networks (FCNs, without fully connected layers) and U-Net (extended to work with fewer training images and to achieve more precise segmentations). The corresponding network structure is shown in Figure 5. In this section, we reviewed all the deep-learning methods for cyst/fluid segmentation in OCT images based on their modification of the original neural network architectures by three subcategories, fine-tuned structure, modified loss function, and adding modules.

**Table 2:** Typical deep learning method for cysts/fluid segmentation.

Architectures	CNN	FCN	U-Net
Fine-tuned structure	Chen et al. [62], Wang et al. [63]	Schlegl et al. [64], Sappa et al. [65]	Roy et al. [66], Kang et al. [67]
Modified loss function	NA	Liu et al. [68], Pawan et al. [69]	Tennakoon et al. [70], Liu et al. [71]
Adding modules/structure	NA	NA	Chen et al. [72], Ma et al. [73]

NA: Not averrable.

**Table 3:** The performance of deep learning method for cysts/fluid segmentation.

Paper	Algorithm	Data Set	Disease	Performance
Chen et al. [62]	Faster R-CNN	RETOUCH dataset	AMD, RVO	DC: 0.997 ACC: 0.665
Wang et al. [63]	OCT-DeepLab	8676 volumes OCT	Macular edema	AUC:0.963
Schlegl et al. [64]	FCN	1200 volumes OCT	AMD, DME, RVO	AUC: 0.94
Sappa et al. [65]	RetFluidNet	124 volumes OCT	AMD	DC: 0.885
Roy et al. [66]	ReLayNet	Duke DME dataset	DME	DC: 0.77
Kang et al. [67]	U-Net	RETOUCH dataset	AMD, RVO	ACC: 0.968, DC: 0.9
Liu et al. [68]	FCN	RETOUCH dataset	AMD, RVO	DC: 0.744
Pawan et al. [69]	DRIP-Caps	25 volumes OCT	CSCR	DC: 0.927
Tennakoon et al. [70]	U-Net	RETOUCH dataset	AMD, RVO	DC: 0.737
Liu et al. [71]	SGNet	Duke DME dataset	DME	DC: 0.8
Chen et al. [72]	SEUNet	UMN dataset	IRF, SRF, PED	DC: 0.9421
Ma et al. [73]	LF-UNet	58 volumes OCT	DME	DC: 0.5132

ACC: accuracy rate; DC: dice coefficient; AUC: area under the curve.

##### CNN and FCN Backbone

Since 2017, various CNN and FCN architectures have been proposed for the purpose of cyst/fluid region segmentation in OCT imaging. FCNs, a subcategory of CNNs, are specifically designed for semantic segmentation tasks, where the objective is to assign a label to each pixel in an image. The structure of FCNs is similar to that of CNNs, yet it employs a unique architecture that maintains the spatial information of the image throughout the network. The utilization of CNNs in image recognition tasks offers several advantages, such as the capacity to automatically learn and extract features from data, the ability to handle high-dimensional data such as images, and the capability to generalize effectively to new data. FCN benefits from the strengths of CNNs and further allows for handling dense prediction tasks, such as semantic segmentation.

Chen et al. [62] used a faster R-CNN to segment different retinal fluids, including the intra-retinal fluid (IRF), sub-retinal fluid (SRF), and pigment epithelium detachment (PED) regions. First, they segmented the IRF regions and then grew the regions to segment the SFT to avoid overfitting. Next, the RPE layer was utilized to segment the PED regions. On the other hand, Schlegl et al. introduced an eight-layer FCN for end-to-end segmentation by utilizing a dataset consisting of 1200 OCT images of patients with AMD, DME, or RVO [64].

In recent years, more complex network structures were applied for the retinal fluid/cyst segmentation. A CNN-based model based on the DeepLab framework was presented by Wang et al. [63] for the segmentation of macular edema in OCT images. The model incorporates atrous spatial pyramid pooling (ASPP) for coarse segmentation and refinement using a fully connected conditional random field (FC-CRF). The proposed method demonstrated superior performance in terms of precision, sensitivity, specificity, and F1-score compared to conventional CNNs. ASPP was also involved in Sappa’s work, RetFluidNet [65]. RetFluidNet architecture integrates skip-connect approaches with ASPP and is capable of segmenting three types of retinal fluids, including IRF, SRF, and PED. The author also involved an investigation of the impact of hyperparameters and skip-connect techniques on fluid segmentation.

In addition to the network structures, revisions of loss functions have also been considered to improve the segmentation performance. Liu et al. used an uncertainty-aware semi-supervised framework to tackle the issue of small fluid area detection and to prevent close regions from being identified as a unified entity. The framework consists of a teacher network and a student network that share a common architecture and employ three decoders to predict the probability map, contour map, and distance map of the image, thus enabling segmentation [68]. In light of the limitations of CNN, Pawan et al. [69] have chosen to utilize the SegCaps architecture [74] based on the capsule networks concept, for segmenting SRF in central serous chorioretinopathy (CSCR) OCT images. To further improve the performance, the authors introduced DRIP-Caps, an enhancement to SegCaps that incorporates the principles of dilation, residual connections, inception blocks, and capsule pooling. The resulting model demonstrates superior accuracy in segmentation, even with limited available sample data.

##### U-Net Backbone

Another area of research in deep learning for segmentation is based on the U-Net architecture. This architecture, a well-established type of FCN, is frequently utilized for the segmentation of medical images. The U-Net design features a contracting path and an expanding path, which facilitates the localization of features with precision and the training of the network in an efficient manner. This unique design has proven to be effective in a variety of biomarker segmentation research applications.

In the study conducted by Roy et al. [66], a novel network called ReLayNet was proposed that combined the concepts of U-Net and DeconvNet and was trained using a joint loss function combining weighted logistic regression and the Dice overlap loss. Kang et al. [67] designed a two-phase neural network for the segmentation of fluid-filled regions, IRF, SRF, and PED. The initial network was aimed at detecting and segmenting fluid regions, while the latter network strengthened the robustness of the former network. The former network took the form of a U-net as the architecture with a classification layer, while dropout and max-out activation were employed to counteract overfitting in training. In the U-Net architecture, modifications were made to the loss function of the neural network. For instance, Tennakoon et al. proposed a method founded on a U-Net architecture, which is trained with a combined loss function incorporating cross-entropy, dice, and adversarial loss terms. The incorporation of adversarial loss enhances the encoding of higher-order relationships between image pixels, obviating the need for a separate post-processing stage for segmentation [70]. Another study by Liu et al. [71] presented a semi-supervised CNN approach for fluid region segmentation in OCT. The architecture consists of two networks, namely a segmentation network and a discriminator network, both of which possess a similar architecture to the U-Net model. The objective function involves a joint loss function that incorporates multi-class cross-entropy loss, dice overlap loss, adversarial loss, and semi-supervised loss. They showed using a discriminator network and unlabeled data can enhance the performance of the fluid segmentation.

Recently, new modules were implemented in the U-Net construction. Chen et al. [72] introduced a network with squeeze-and-excitation blocks accruing to U-Net named SEUNet for the segmentation of fluid regions and classifying OCT to AMD or normal image. The steps of SE-block, squeeze, excitation, and scale can evaluate the degree of importance of the feature map of the image and then enhance the helpful feature according to the degree of importance. Ma et al. [73] proposed an architecture called LF-uNet for the retinal layer and cyst/fluid segmentation that integrates elements, such as the uNet block, FCN block, multi-scale ASPP, parallel inception block, and cascaded order.

## 3. OCT Datasets for Cyst/Fluid Segmentation

**Figure 6:**
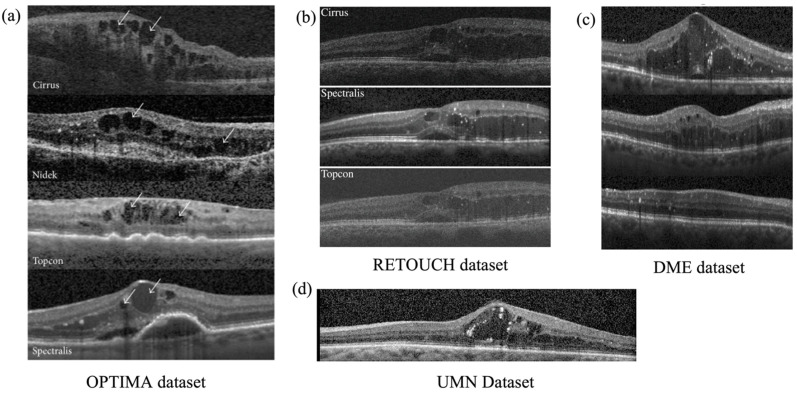
Sample OCT images from public datasets: (**a**) OPTIMA dataset [75]; (**b**) RETOUCH dataset [76]; (**c**) DME dataset [52]; (**d**) UMN dataset [77].

The process of training a cyst/fluid segmentation model requires the use of annotated OCT datasets, where the location of fluid in each image is labeled by experts. This labeling process is labor-intensive, which is why there are numerous public OCT datasets available for use. Table 4 provides a list of well-known public datasets that pertain to OCT and Figure 6 presents a sample illustration.

### 3.1. The OPTIMA Dataset

The OPTIMA dataset [75] was made available to the public through the cyst segmentation challenge at the Medical Image Computing and Computer-Assisted Intervention (MICCAI) 2015 conference and has since become a commonly used resource for IRF segmentation in OCT. The dataset consists of 30 volumes of patient data obtained from various OCT devices, including Spectralis, Topcon, Cirrus, and Nidek. Each retinal OCT volume has dimensions approximately 6 × 6 × 2 mm^3^ and is centered on the macula. The dataset has been further divided into 15 training and 15 testing volumes, which have been chosen to reflect the broad range of scans encountered in clinical settings, as well as the diverse appearances and distributions of IRF. The IRF in the training data was annotated by two expert graders at the Medical University of Vienna.

### 3.2. The RETOUCH Dataset

The RETOUCH dataset [76] was derived from the retinal OCT fluid challenge of the MICCAI conference in 2017, where the OCT images were annotated with labels of retinal fluid: IRF, SRF, and PED. The dataset consists of 70 OCT volumes acquired from patients diagnosed with macular edema secondary to AMD or RVO. Of these volumes, 24 were obtained from the Cirrus OCT system, 22 from the Triton OCT system, and 24 from the Spectralis OCT system. The annotations were made by human graders supervised by retinal specialists at the Medical University of Vienna and Radboud University Medical Center in the Netherlands. The B-scan images acquired by the Cirrus, Triton, and Spectralis OCT systems contain 128, 128, and 49 images, respectively, with each image having a different resolution. The RETOUCH dataset has been widely used as a benchmark for evaluating the performance of retinal cyst/fluid segmentation algorithms and has been the basis for many relevant studies in this field.

### 3.3. DME Dataset from Duke

The DME dataset [52], made available by the Vision and Image Processing Laboratory at Duke University, is a well-known public resource comprising 110 annotated B-scan images from patients with severe DME pathology. The images were acquired with a primary focus on the fovea, with supplementary five frames acquired laterally at distances of ±2, ±5, ±10, ±15, and ±20 μm from the foveal slice. The boundaries of the eight retinal layers, as well as fluid and non-fluid regions, were annotated by two fellowship-trained medical retina specialists at the Duke Eye Center.

### 3.4. UMN Dataset

The UMN dataset [77] was gathered by the University of Minnesota Ophthalmology Clinic, consists of 600 OCT B-scan images obtained from 24 patients diagnosed with exudative AMD. The images were captured using the Spectralis system, with each patient undergoing approximately 100 B-scans. From these, 25 images with the largest fluid area were selected as the samples. The retinal fluid, IRF, SRF, and PED were annotated and verified by two ophthalmologists.

## 4. Discussion

### 4.1. OCT Application and Cyst Segemation in Ophthalmology

OCT is a non-invasive imaging technique that utilizes the principle of interferometry to produce cross-sectional views of the retina. The technology has a resolution capability of at least 10–15 microns, which has led to its widespread adoption as an ophthalmic diagnostic tool. OCT was first introduced in 1991 and has since been used to image various ocular conditions, including DR and AMD. The versatility of OCT has extended beyond ophthalmology, as it has been utilized to image non-transparent tissues in other fields. In ophthalmology, it has become a crucial tool in the diagnosis, prognosis, and management of various ocular conditions. The non-invasive imaging capability of OCT to produce detailed images of the retina non-invasively has revolutionized the way ocular conditions are diagnosed and treated and has facilitated early detection and effective management of conditions. OCT’s precise and reliable images have made it a critical tool in advancing ocular medicine. Its ability to non-invasively image the retina has enabled a greater understanding of retinal structure and function, leading to improved patient outcomes.

**Figure 7:**
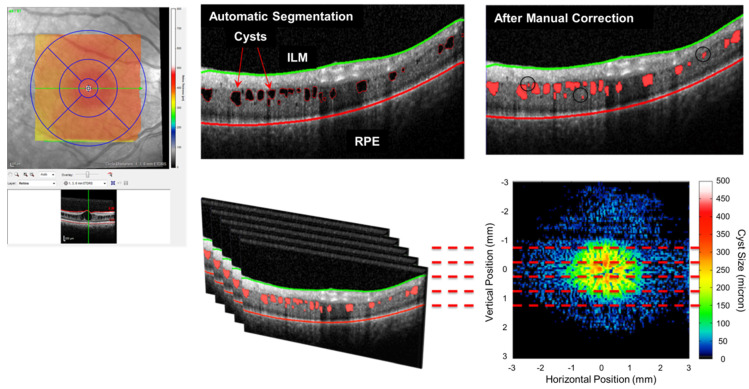
An application of using cyst segmentation in OCT for a patient with XLRS. The figure is reprinted with permission from Ref. [78]. Pennesi, 2018.

The implementation of an automatic cyst/fluid segmentation system has the potential to greatly aid ophthalmologists in their research endeavors. For example, in a study conducted by Pennesi. et al. [78], an automatic cyst/fluid segmentation system was utilized in the context of X-linked retinoschisis (XLRS) patients. The automated layer segmentation method was applied to detect the ILM and RPE, and identify dark regions between these two structures. From these dark regions, a neural network was trained using the features including intensity, shape, and position to classify the region as cyst or not (as shown in Figure 7). The ability to calculate the volume of cysts fast using this automated system provides a valuable tool for conducting follow-up studies in XLRS patients.

### 4.2. The Connection between Image Denoising, Layer Segmentation, and Cyst Segmentation

For the methods we reviewed, the classical machine learning or statistic-based methods require human-designed features. Still, they have the benefits of explainability for results and flexibility for a small dataset. The deep-learning-based method is more powerful but data-hungry. The cyst segmentation framework has three components: image denoising for removing the speckle in OCT, layer segmentation for finding the research region of the cyst, and cyst segmentation for finding the cyst region. These three parts are designed independently. The perspective of our review provides a research direction to test each possible combination of these components and find the most suitable method in each step. On another hand, with the benefits of deep learning, numerous works are designed as an end-to-end system, which only includes one neural network to handle tasks from all three components. They need fewer computational resources and avoid the error propagation from the layer segmentation to the cyst segmentation. In the current situation, no specific evaluation exists to determine which types of methods are superior and which should be investigated in the future.

### 4.3. Performance of Classical Machine Learning and Deep Learning

We presented a comparison of the performance of classical machine learning methods and deep learning algorithms. Table 1 and Table 3 summarize the performance of these algorithms. However, comparing these algorithms’ performance is challenging due to the variations in datasets and evaluation criteria used in different studies. The most frequently used metrics for evaluating the performance of segmentation algorithms are the accuracy rate (ACC) and dice coefficient (DC). ACC measures the proportion of correctly classified pixels or voxels, while DC evaluates the agreement between the segmented regions and the ground truth by measuring the overlap between the two regions. DC is commonly used in medical imaging applications and ranges between 0 and 1, where 1 indicates perfect overlap and 0 indicates no overlap. Other metrics, such as the area under the curve (AUC), true positive volume fraction (TPVF), false positive volume fraction (FPVF), and relative volume difference ratio (RVD), are also used to assess the performance of segmentation algorithms. AUC is a widely used performance metric for binary classification models and measures the model’s ability to distinguish between positive and negative classes by computing the area under the receiver operating characteristic (ROC) curve. TPVF and FPVF measure the proportion of correctly and incorrectly classified pixels or voxels. RVD is a metric that assesses the accuracy of volume measurement by calculating the percentage difference between the measured volume and the true volume of the target region.

Classical machine learning approaches, such as González [48] and Gopinath [49], achieved accuracies of 80% and 76.7% using SVM and random forest algorithms. Quellec et al. [27] reported an AUC of 0.961 using K-nearest neighbor algorithms. On the other hand, Chen [55] and Zhu [56] evaluated their algorithms using TPVF and FPVF, with Chen achieving TPVF of 86.5% and FPVF of 1.7%, while Zhu achieved TPVF of 81% and FPVF of 0.80%. Chiu [52] achieved a dice coefficient of 0.809 using kernel regression on public data from Duke, comparable to deep learning algorithms ReLayNet [66] of Roy et al. (0.77) and SGNet [71] of Liu et al. (0.8).

In the domain of deep learning, researchers commonly favor the utilization of public datasets, with the RETOUCH dataset being the prevailing choice. Among the deep learning algorithms evaluated in this study, faster R-CNN by Chen et al. [62], modified U-net by Kang et al. [67], modified FCN by Liu et al. [68], and revised U-net by Tennakoon et al. [70] were applied on the RETOUCH dataset and achieved dice coefficients of 0.997, 0.9, 0.744, and 0.737, respectively. In contrast, Wang et al.’s OCT-DeepLab framework [63] and Schlegl et al.’s FCN [64] relied on private data and achieved AUC values of 0.963 and 0.94, respectively. Notably, Chen et al.’s SEUNet [72], based on the U-net architecture and leveraging the public UMN dataset, attained a dice coefficient of 0.9421. Furthermore, Sappa et al.’s RetFluidNet [65], utilizing private data from AMD patients, yielded a dice coefficient of 0.885, while Pawan et al.’s DRIP-Caps algorithm applied to data from patients with CSCR, achieved a dice coefficient of 0.927. Lastly, Ma et al.’s LF-UNet [73] was employed on a private dataset of patients with DME and produced a dice coefficient of 0.5132.

### 4.4. Challenges, Opportunities, and Future Direction

The advances in AI/machine learning and its applications in ophthalmology have brought significant benefits, but also present several limitations. One of the limitations is the lack of integration of domain knowledge in current OCT cyst segmentation algorithms. Typically, deep neural networks are trained using large amounts of supervised data. However, manual annotation of these datasets is a tedious and time-consuming process, particularly in the medical field where specialized experts are required. For cyst segmentation, doctors have concrete domain knowledge and rules to distinguish the cyst area in the OCT. However, most current works ignore this part, and the whole learning structure is designed purely from raw data. So, encoding this knowledge is a more scalable solution which potentially allows for finding better models. The techniques can be involved in the design of the algorithms: Bayesian-style learning method and knowledge-based learning.

Another limitation is the volume and diversity of data available for training. Existing machine learning algorithms are highly data-dependent, and while publicly annotated cyst datasets are available, they are not enough to train powerful neural networks. As a result, techniques, such as transfer learning and few-shot learning, should be employed to enhance the size and quality of training datasets. Furthermore, current datasets are often lacking in representation of diverse ethnicities and may not be inclusive of all relevant features. To maximize the generalizability and applicability of deep learning algorithms, it is important to train these algorithms on data obtained from various hardware devices and not limit them to a specific one. As the application of AI in healthcare continues to grow, the exchange of medical information between patients and physicians through it is becoming increasingly prevalent. This highlights the importance of balancing data privacy and data utilization efficacy. One solution to this challenge is the use of federated learning, which enables AI institutions/hospitals to collaborate in a cooperative manner, utilizing data from multiple sources while avoiding privacy issues. Federated learning is a novel approach to training machine learning algorithms, which diverges from the centralized transactional approach. In federated learning, algorithms are trained on multiple decentralized servers/hospitals, each containing local data samples, without the need for data exchange between servers/hospitals. This novel approach to machine learning provides a solution to the data privacy and data utilization efficacy challenges.

Lastly, current OCT cyst segmentation algorithms lack explanation and trustworthiness, which is a critical issue in fields affecting people’s health. It is imperative to understand the reasoning behind these decisions, as this could provide insight into the rules used by the models and prevent potential unintended consequences. Thus, the development of human-understandable explanations for these models is a crucial aspect of the practical implementation of artificial intelligence in healthcare. In the case of the cyst and fluid segmentation, providing explanations of the segmentation results can increase the trustworthiness of the recognition results. Most of them are viewed as black boxes with unknown mechanisms. It is essential to understand why decisions are made, which helps us understand the rules the models use to make decisions and prevent potentially unexpected situations from happening. Generating human-understandable explanations for the models is an essential topic for using AI in the real world. In the cyst/fluid segmentation, the explanation of the segmentation made the recognition results more trustworthy for doctors.

## 5. Conclusions

OCT images are increasingly used in the diagnostic and prognostic of retinal disease. It is essential to evaluate retinal cysts/fluid for the purpose of determining initial diagnosis and retreatment. However, the manual evaluation of large OCT datasets is time-consuming and error prone. In order to effectively process the increasing amount of images generated by modern OCT devices and provide accurate cyst or fluid detection, automated learning-based methods are required. Ideally, the method should not be restricted to one disease or device and could be applied to a wide range of diseases.

In this study, we reviewed the typical existing works in OCT cyst/fluid segmentation, while emphasizing the significance of machine learning applications. We delve into the three fundamental steps of the problem, including image denoising, layer segmentation, and cyst segmentation, highlighting the most prominent state-of-the-art algorithms for each step and comparing their efficacy. Moreover, the challenges, opportunities, and potential avenues for future growth in the application of AI in OCT cyst segmentation were also explored. The insights gained from this review can serve as a valuable resource for those seeking to apply the current segmentation technique for disease diagnosis or advance the field through novel algorithmic designs.

## Figures and Tables

**Table 1:** Classical machine learning methods for cyst segmentation.

Paper	Core Method	Preprocessing	Retinal Layer Segmentation	Cyst Segmentation	Post-Processing	Disease	Data	Performance
Quellec et al. [27]	K-nearest neighbor [40]	Wavelet transform [15]	Multiscale 3-D graph search technique [47]	K-nearest neighbor	None	AMD	91 macula-centered 3-D OCT volumes	AUC: 0.961
González [48]	Support vector machine [41]	None	ILM RPE	Watershed algorithm [20] naive-Bayes, SVM, random forest	Discarding invalid candidate regions by properties: size, location, etc.	DR	30 HD-OCT retinal gray-scale images	ACC: 80%
Gopinath [49]	Random forest [42]	Image size standardized. Total variational denoising [50]	Graph theory-based segmentation approach [33]	The center-surround difference method [51], random forest	None	NA	15 volumes of SD-OCT scans: MICCAI 2015	ACC: 76.7%
Chiu [52]	Kernel regression [43]	DME algorithm [53,54], KR-based denoising	ILM and BM	KR-based kernel regression	None	DR	Duke Enterprise Data Q = 61 B-scans × N = 768	DC: 0.79
Chen [55]	Graph search-graph cut [44]	SVM	11-surface segmentation	GS-GC method	DME	NA	15 spectral domain OCT images	TPVF: 86.5%, FPVF:1.7%, RVDR: 12.8%
Zhu [56]	AdaBoost [45]	A SNR balancing, A 3-D curvature anisotropic diffusion filter	Multiscale 3-D graph search technique [47]	AdaBoost classifier	Macular edema, MH	NA	SD-OCT scan of 19 eyes with coexistence of CMEs and MH from 18 subjects.	TPVF: 81%, FPVF: 0.80%, ACC: 99.7%, DC: 0.809

TPVF: true positive volume fraction; FPVF: false positive volume fraction; ACC: accuracy rate; DC: dice coefficient; AUC: area under the curve; RVDR: relative volume difference ratio; NA: Not available.

**Table 4:** Public OCT datasets for retinal cyst/fluid segmentation.

Dataset	Dataset Size	Fluid/Cyst Type	OCT Vendor	Disease
OPTIMA [75]	30 volumes	IRF	Spectralis, Topcon, Cirrus, Nidek	AMD, RVO, DME
RETOUCH [76]	70 volumes	PED	Spectralis, Topcon, Cirrus, Nidek	AMD, RVO
Duke DME [52]	110 B-scan images	All fluid-filled regions	Spectralis	DME
UMN [77]	600 B-scan images	IRF, SRF, PED	Spectralis	AMD

## Data Availability

Not applicable.

## Abbreviations

The following abbreviations are used in this manuscript:

ACC	Accuracy rate
AI	Artificial intelligence
AMD	Age-related macular degeneration
ASPP	Atrous spatial pyramid pooling
AUC	Area under the curve
CL	Connecting cilia
CME	Cystoid macular edema
CNN	Convolutional neural network
DC	Dice coefficient
DR	Diabetic retinopathy
FC-CRF	Fully connected conditional random field
FCN	Fully Convolutional Network
FOM	Figure of merit
FPVF	False positive volume fraction
GCL	Ganglion cell layer
GCN	Graph convolutional network
GS-GC	Graph search-graph cut
INL	Inner nuclear layer
IPL	Inner plexiform layer
IRF	Intra-retinal fluid
ISL	Inner segment layer
k-NN	K-nearest neighbor
LDF	Learnable de-speckling framework
MH	Macular hole
MICCAI	Medical Image Computing and Computer-Assisted Intervention
MS	Multiple sclerosis
MSBTD	Multiscale sparsity-based tomographic denoising
NFL	Merve fiber layer
OCT	Optical coherence tomography
OLM	Outer limiting membrane
ONL	Outer nuclear layer
OPL	Outer plexiform layer
OSL	Outer segment layer
PED	Pigment epithelium detachment
RNN	Recurrent Neural Network
RPE	Retinal pigment epithelium
RVD	Relative volume difference ratio
RVO	Retinal vein occlusion
SAWF	Spatially adaptive wavelet filter
SNR	Signal-to-noise ratios
SRF	Sub-retinal fluid
SVM	Support vector machine
TPVF	True positive volume fraction
VM	Verhoeff’s membrane
XLRS	X-linked retinoschisis
